# Investigation on Flexural Fracture Behaviour of Bolted Spherical Joints with Crack Propagation in Screw Threads

**DOI:** 10.3390/ma16103781

**Published:** 2023-05-17

**Authors:** Qinghong Shi, Wenfeng Zhou, Xiang You, Yinggai Liu, Zhiyu Wang, Qunyi Huang

**Affiliations:** 1 Key Laboratory of Deep Underground Science and Engineering (Ministry of Education), School of Architecture and Environment, Sichuan University, Chengdu 610065, China; 2 CREGC Architectural & Construction Engineering Co., Ltd., Chengdu 610031, China; 3 College of Civil Engineering, Southwest Jiaotong University, Chengdu 610031, China

**Keywords:** fracture behaviour, bolted spherical joints, flexural bending, numerical modelling, fracture mechanics

## Abstract

Bolted spherical joints, due to their prominent merits in installation, have been widely used in modern spatial structures. Despite significant research, there is a lack of understanding of their flexural fracture behaviour, which is important for the catastrophe prevention of the whole structure. Given the recent development to fill this knowledge gap, it is the objective of this paper to experimentally investigate the flexural bending capacity of the overall fracture section featured by a heightened neutral axis and fracture behaviour related to variable crack depth in screw threads. Accordingly, two full-scale bolted spherical joints with different bolt diameters were evaluated under three-point bending. The fracture behaviour of bolted spherical joints is first revealed with respect to typical stress distribution and fracture mode. A new theoretical flexural bending capacity expression for the fracture section with a heightened neutral axis is proposed and validated. A numerical model is then developed to estimate the stress amplification and stress intensity factors related to the crack opening (mode-I) fracture for the screw threads of these joints. The model is validated against the theoretical solutions of the thread-tooth-root model. The maximum stress of the screw thread is shown to take place at the same location as the test bolted sphere, while its magnitude can be greatly reduced with an increased thread root radius and flank angle. Finally, different design variants related to threads that have influences on the SIFs are compared, and the moderate steepness of the flank thread has been found to be efficient in reducing the joint fracture. The research findings could thus be beneficial for further improving the fracture resistance of bolted spherical joints.

## 1. Introduction

**Figure 1:**
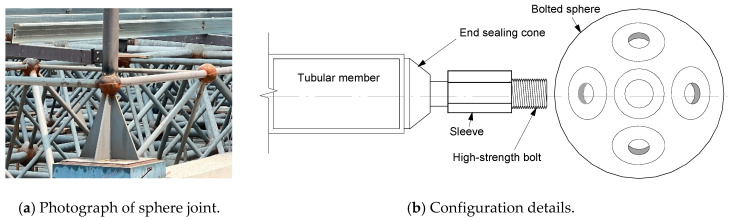
Illustration of a typical bolted spherical joint.

Bolted spherical joints, owing to their high installation accuracy and flexible joining directions [1], have become a promising choice for architects and engineers in the construction of long-span spatial structures, such as exhibition halls and metro spaces [2]. Taking a typical joint configuration, for instance, as shown in Figure 1, a tubular member with an end sealing cone is joined to a bolted sphere through a driven high-strength bolt with the sleeve screwed. The load transfer of the bolted spherical joint is primarily dependent on the high-strength bolted connection, and its deficiency would induce fracture of the joint and even the catastrophe of the whole spatial structure.

Extensive research work has therefore been conducted to understand the damage mechanism of the bolted spherical joints under various loading conditions. For example, Piroglu et al. [3] analysed a partially collapsed steel space truss roof structure in terms of conformity checking of the structural members and discontinuously heated roof parts after exceptional snowfall. Feng et al. [4] and Xiong et al. [5] investigated the failure modes of single-layer reticulated shells affected by the bending stiffness of bolted spherical joints affected by semi-rigidity. Yan et al. [6] investigated the looseness of bolt-sphere joints in steel grid structures and established a relationship between ultrasonic energy attenuation and the bolt loosening level. Liu et al. [7] conducted a series of tests on the tensile failure of postfire bolt-sphere joints influenced by material type, high temperatures, and cooling modes. Yuan et al. [8] simulated the loading performance degradation of pitted bolt-sphere joints with the incorporation of stochastic corrosion pits. Furthermore, the importance of the high-strength bolt for the load-carrying capacity of the bolted spherical joints can be found in the recent literature. Ma et al. [9] concluded from a simulation that the stiffness and ultimate strength of the joint can be significantly improved by the central hollow hexagonal prism and front bolts. Lei et al. [10] found that the damage to the grid structure tends to cause fatigue fracture at high-strength bolt joints where the threads result in a significant stress concentration leading to bolt fatigue. Wu et al. [11] showed the effect of an insufficient bolt screwing depth on the mechanical behaviour of the bolted spherical joints and the stability of the single-layer reticulated shell. Similar effects of bolts on the behaviour of bolted spherical joints under fire and conditions can be found in Refs. [12,13,14], respectively.

Although the details of high-strength bolts for the configuration of space grid structures have been codified in the code of JGJ 7-2010 [15], a lot of attention has been paid to the bolt fracture, including basic geometry [16], heat treatment [17] and grain alignment [18]. The behaviour of the bolted joints is dependent on the stress concentration in the threads. This is especially the case for the failure of the threads when the stresses induced by unequal and fluctuating loading are unevenly distributed with crack initiation and propagation. An appropriate design of the shape of the bolt to decrease the stress concentration at the root of loaded threads is therefore of great interest to current researchers. For example, Govindu et al. [19] compared several stress distribution modes in Buttress and ACME threads for various symmetrical models, such as with or without a groove on the bolt or a reduced diameter, to reduce the stress concentration in threads. Van Wittenberghe et al. [20] experimentally studied the standard API line pipe coupling and two modified coupling configurations of threads as stress raisers. The improvement of fatigue life through the coupling’s global geometry is also focused. Mushtaq and Sheikh [21] conducted an experimental evaluation of the effect of preload on the fatigue life of bolts. It was found that the preload reduces the endurance limit of bolts by an amount proportional to the increase in the mean stress produced by the preload, while the stress concentration factor was not affected by the axial load applied to the bolt. Wentzel and Huang [22] performed experimental research on the bending fatigue strength of threaded fasteners. It was found that the fatigue strength was determined by the randomly distributed defects in the highly stressed screw thread root. Recently, Vijay et al. [23] numerically compared stress concentration factors of threaded fasteners, in which the Whitworth threads were found to show a more effective reduction in stress concentration factor, while the square and metric threads showed less.

The fracture mechanics theories have also been applied to the analysis of the crack tip stresses at bolt threads through the calculation of the stress intensity factor (SIF) and the assessment of crack growth. For example, Brennan and Dover [15] reported a multiple reference state weight function theory-based SIF equation for threaded connections that was limited by mode I crack emanating from the screw thread root and neglected the effect of helix angle. Afterwards, Brennan and Dover [24] and Zhao et al. [25] gave a SIF estimation for a surface crack at the screw thread root of a screwed pipe joint based on an axisymmetric bolt model with similar analytical limitations. Shahani and Habibi [26] predicted a mixed SIF for a hollow cylinder subjected to axial force, bending moment, and torsion. A non-symmetrical distribution of stress intensity factors was found along the crack front, causing non-symmetric fatigue crack propagation. Kumar and Prakash [27] formulated a SIF solution for threads in a bolt having a helix angle and a part-through crack emanating from a metric threaded bolt. The effect of crack depth ratios and aspect ratios on SIFs was taken into consideration. Recently, Aliakbari and Mamaghani [28] studied the SIFs of the cylinder head bolts of a four-cylinder gasoline engine imposed by premature failure. The SIF on the short crack length was confirmed to be much more important than the SIF in longer cracks, while the crack existence to a depth of 0.35 mm was the failure source of premature fracture. However, the novelty of the solution of SIF represents a limit to its application for bolted spherical joints because no design equations properly allowing for relevant variants are included in common standards or recent studies in the literature.

Despite significant research work reported in the literature, understanding the fracture behaviour of the bolted spherical joints is still lacking. To bridge this gap, an extensive study is presented in this paper to gain further insight into the flexural bending capacity related to the overall fracture section and the fracture stress intensity factor of the joints with variable crack depth in screw threads. Firstly, two types of bolted spherical joints with different bolt diameters were experimentally examined in terms of stress distributions and fracture modes when subjected to flexural bending. A theoretical expression for predicting the flexural bending capacity of the fracture section allowing for a heightened neutral axis is proposed and validated. A numerical model is then developed to estimate the critical stress gradients near the screw thread root. The modelling results are also validated against the theoretical calculation when the cracks are initiated and propagated from the screw thread root. The geometric parameters related to the SIFs of the crack propagated from the screw thread root to the bolt shank body are explored and discussed.

## 2. Experimental Program

### 2.1. Specimen Description

**Figure 2:**
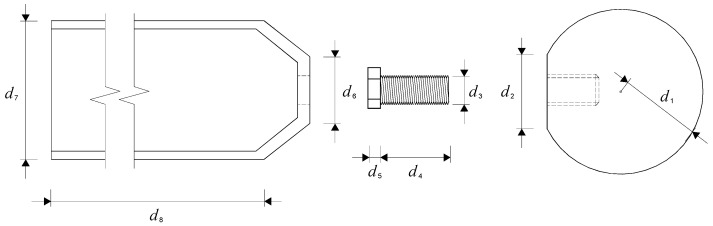
Schematic dimension of the bolted spherical joint.

For the sake of studying the fracture properties of the bolted spherical joints under flexural bending, two types of joints with different high-strength bolts (i.e., M20 and M24) as standard fittings for steel grid structures were selected. The test specimens were also designed according to the Technical Specification for Space Grid Structures (JCJ7-2010) [15]. In actual practise, the high-strength bolt will sustain a proportion of the applied loading prior to the joint separating. The basic parameters of the tested bolted spherical joints are shown in Table 1 and Figure 2. The material properties for the high-strength bolt were obtained from tension tests conducted on three coupons, while those for the bolted sphere, the sleeve, and the end sealing cone were referred to in the report provided by the producer. Each of the tested bolted spherical joints consists of two 10.9-grade high-strength bolts and one 355 grade bolt-sphere with a radius of 50 mm. The screwing depths measured by a digital vernier calliper were chosen as 0.95~1.05 times the diameter of the high-strength bolts to ensure that the shear capacity of the threads is considerably higher in contrast to the tensile capacity of the bolt shank. Although the sleeve in the bolted spherical joints was neglected due to the unloading effect in the literature focusing on uniaxial tension behaviour [11,14], it was still taken into account in this study since its loading stress from the end sealing cone cannot be ignored in the upper region in compression produced by flexural bending. The material mechanical properties for the high-strength bolt, the bolted sphere, the sleeve, and the end sealing cone are defined in isotropic and linear elastic relations as listed in Table 2.

### 2.2. Test Setup and Procedure

**Figure 3:**
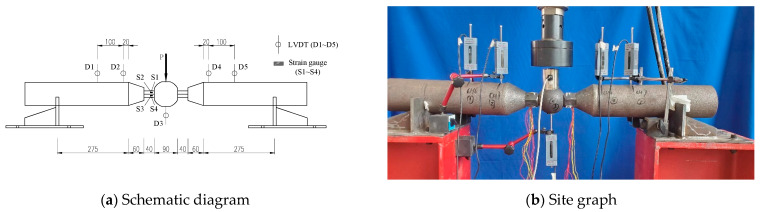
Test setup.

The bolted spherical joints with tubular members were tested in three-point flexural bending following a test setup as sketched in Figure 3. The constituent tubular members rest on two rigid supports laid on a rigid base frame, permitting rotation of the tubular members. The flexural bending tests on the bolt-sphere joints with tubular members were carried out on a 30 t vertical actuator. Firstly, the high-strength bolts through the end sealing cones and the sleeves were screwed into the bolted sphere, and the depth of the bolt screwed into the bolted sphere was ensured. Finally, the supports and loading point were fixed to the testing rig to complete the installation of the test specimen. The load cell was mounted below the actuator for which the load applied to the test specimen (denoted by *P*) was measured. The flexural moment can then be converted by multiplying the span length, *L*_0_, as *M* = 0.25*PL*_0_. During the experiment, the load was first applied using load control at a speed of 0.5 mm/min until the yield strength of the high-strength. It is expected that if the crack initiates and propagates at the end of this stage, it will generate an increasing stress intensity factor at the crack tip, causing the material to fracture. Thereafter, the load application was switched to displacement control at multiple yield displacements to stabilise or even arrest subsequent cracking. The test was stopped until the bolted spherical joint was damaged or the load carrying capacity was significantly reduced.

The load-point displacement is measured in relation to points on the upper surface of the tubular members. Since it is easier to measure the displacement due to the dimensions of the test specimen, four linear variable differential transducers (LVDTs) fixed onto both sides of the bolted spherical joint were used to measure the displacement of the tubular members. Considering the distance between LVDT D1 and LVDT D2 and that between LVDT D4 and LVDT D5 are denoted by *L*_1,2_ and *L*_3,4_, respectively, the rotation of the bolted spherical joint can be calculated as *θ* = 0.5atan[(*δ*_2_ − *δ*_1_)/*L*_1,2_] + 0.5atan[(*δ*_5_ − *δ*_4_)/*L*_4,5_. Additionally, a LVDT installed below the central loading of the bolted spherical joint measured the loading-point displacement relative to the frame. Additionally, four strain gauges labelled S1~S4 were mounted consecutively from the upper to the bottom height of the sleeve. The ultimate load-carrying capacities and load-displacement curves of the bolted spherical joints under different degrees of deflection were recorded during the loading progress.

## 3. Theoretical Basis Related to Flexural Bending and Fracture

As concerns the load carrying capacity, both test specimens broke with crack propagation of the bolt shank from the screw thread root subsequent to flexural bending. It is necessary to understand the flexural bending capacity of the fracture section close to the contact surface between the bolted sphere and the sleeve. A theoretical basis is also presented for the fracture-related stress intensity factor for the test bolted spherical joints in what follows.

### 3.1. Flexural Bending Capacity

**Figure 4:**
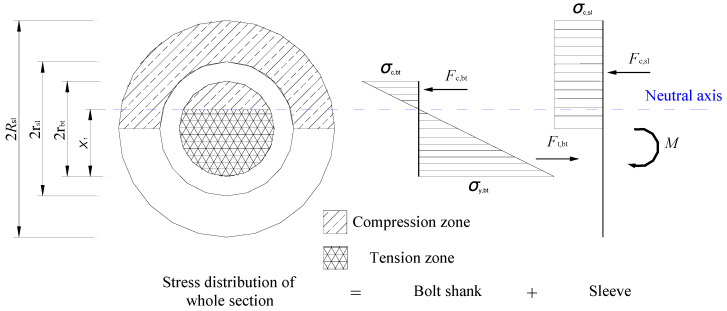
Stress distribution diagram for the fracture cross section of a bolted spherical joint.

For purposes of generality, the theory for the flexural bending capacity of the test bolted spherical joints is derived based on the following assumption: (1) the material model of all joint components follows elastic-perfectly plastic relations; (2) the sleeve and the bolt shank are deformed with desired compatibility; (3) a plane remains plane during bending for the combined section containing the bolt shank and the sleeve; (4) the geometry of the fracture section is defined by the radius of the bolt shank (*r*_bt_) and the internal radius (*r*_sl_) and external diameter (*R*_sl_) of the sleeve, as shown in Figure 4.

When the test bolted spherical joints are subjected to continuous flexure, the sleeve in the bottom tension zone of the joint is separated from the bolted sphere. Meanwhile, the upper parts of the sleeve and the bolt shank are in compression. As a result, the neutral axis of the section is heightened away from the bottom edge of the bolt shank, and its relative distance is denoted by *X*_1_. Following the upper edge of the sleeve steps into the plasticity, the compressive zone of the sleeve is growing until the whole sleeve section above the horizontal centroidal axis can be defined by its corresponding yield stress and the tensile resultant force as *σ*_c,sl_ and *F*_c,sl_, respectively. Furthermore, the normal stress varies linearly over the cross section of the bolt shank, with an elastic limit in tensile stress (*σ*_y,bt_) equal in magnitude to that in compressive stress (*σ*_c,bt_). Similarly, the tensile and compressive resultant forces for *σ*_y,bt_ and *σ*_c,bt_ can be denoted as *F*_t,bt_ and *F*_c,bt_, respectively.

Based on the section shown in Figure 3, the resultant forces, *F*_t,bt_, *F*_c,bt_, and *F*_c,sl_, can be expressed in integral expressions with respect to the distance to the horizontal centroidal axis, *x*, as follows:

(1) $$F_{t,bt}=\int_{-r_{bt}}^{X_{1}-r_{bt}}\left[-\frac{\sigma_{y,bt}}{X_{1}}x+\frac{\sigma_{y,bt}}{X_{1}}\left(X_{1}-r_{bt}\right)\right]\cdot 2\sqrt{r_{bt}^{2}-x^{2}}dx$$

(2) $$F_{c,bt}=\int_{X_{1}-r_{bt}}^{r_{bt}}\left[-\frac{\sigma_{y,bt}}{X_{1}}x+\frac{\sigma_{y,bt}}{X_{1}}\left(X_{1}-r_{bt}\right)\right]\cdot 2\sqrt{r_{bt}^{2}-x^{2}}dx$$

(3) $$F_{c,sl}=0.5\pi \left(R_{sl}^{2}-r_{sl}^{2}\right)\sigma_{y,sl}$$

Because there is an equilibrium of the resultant forces in the horizontal direction of the whole cross section, the following formulation can be written as follows:

(4) $$F_{t,bt}=F_{c,bt}+F_{c,sl}$$

Substituting Equations (1)–(3) into Equation (4), *X*_1_ can be obtained as follows:

(5) $$X_{1}=\frac{2\sigma_{y,bt}r^{3}}{2\sigma_{y,bt}r^{2}-\sigma_{y,sl}\left(R_{sl}^{2}-r_{sl}^{2}\right)}$$

The flexural bending capacity of the fracture section, allowing for a heightened neutral axis, can then be determined using the lever arm in relation to the distance of the constituent parts to the upper edge of the sleeve as follows:

(6) $$M_{pred}=\frac{\sigma_{y,bt}}{X_{1}}\left[0.25\pi r_{sl}^{4}+\pi R_{sl}r_{bt}^{2}\left(X_{1}-r_{bt}\right)\right]-0.5\pi \sigma_{y,sl}\left(R_{sl}^{2}-r_{sl}^{2}\right)\cdot \left[R_{sl}-\frac{4\left(R_{sl}^{2}+r_{sl}^{2}+R_{sl}r_{sl}\right)}{3\pi \left(R_{sl}+r_{sl}\right)}\right]$$

A direct comparison of flexural bending moments between the experimental results (*M*_test_) and the theoretical values (*M*_pred_) is listed in the last two columns of Table 1. It can be seen that by using the proposed Equation (6), one could estimate the flexural bending moment of bolted spherical joints to a respectable degree of accuracy, with the percent error in contrast being no more than 5%.

### 3.2. Fracture Stress Intensity Factor

The fracture of the threads of bolted spherical joints is mostly related to the crack opening mode, i.e., mode I, where the displacement at the lips of the crack is perpendicular to the crack propagation. The elasticity plane for the fracture mode can be derived using the Westergaard solution, *Z*, depending on the crack shape and the load type, as documented in Ref. [29], as follows:

(7) $$Z=\frac{f(z)}{\sqrt{z-a}}$$

where *f*(*z*) is series in complex field as *f*(*z*) = *a*_0_ + *a*_1_(*z* − *a*) + *a*_2_(*z* − *a*)^2^ + *a*_3_(*z* − *a*)^3^ + … while *z* = *x* + *iy*. Taking *K*_I_ as the stress intensity factor depending on the stress and crack geometry, *Z* can be singular with *a*_0_, *a*_1_, *a*_2_, … as constants. By convention,

(8) $$\frac{K_{I}}{\sqrt{2\pi }}=\underset{z\to a}{\lim}Z\sqrt{z-a}$$

**Figure 5:**
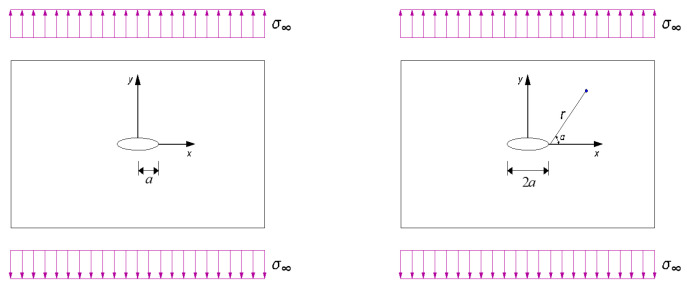
Infinite linear elastic plates with mode I cracks.

For two-dimensional medium loaded in mode I, *K*_I_ in mode I can be determined by the development around *z* = ± *a* in real values for finite pieces with cracks in practical application as follows:

(9) $$K_{I}=\sigma \sqrt{\pi a}\cdot Y(a)$$

where *Y*(*a*) is a dimensionless geometric correction factor depending on the geometry of the piece and the crack length. *Y*(*a*) becomes close to 1.0 when the crack is very small relative to the dimension of the piece; otherwise, it can be correlated as a function of geometry and the crack length *a* and the stress tensor 𝜎_∞_ at a distance with a half crack length, as illustrated in the left side of Figure 5. For an infinite linear elastic plate, only the mode I crack with the circle of radius, *r*, near the crack tip being close to zero is considered, as shown on the right side of Figure 5. The crack front in the linear elastic domain can be characterised by finite element (FE) modelling. The original formulation in the elastic cracked plane for integral *J* in the absence of loading on the crack can be expressed with respect to the elastic strain energy density, *W*, and the traction vector, *T*, as follows:

(10) $$J=\int_{C}Wdy-\bar{T}\cdot \partial \bar{u}/\partial x\cdot dS$$

where *u* is the displacement vector. *C* is the counterclockwise contour initiating at the lower crack surface and terminating on the upper crack surface. The integral is done on any continuous *C* following the fracture tip of the interior crack to the edge of the superior crack. *T* is the friction vector on *C*, which is directed depending on the normal vector to *C* [29].

The geometric correction factor for smooth and notched round bars was suggested by Lefort [30] in the formulation as follows:

(11) $$\begin{array}{l} Y(a)=-3.519+1.361/\left(1-2a/D\right)+0.053/{\left(1-2a/D\right)}^{2}+10.23/\left(1-2a/D\right)\\ -15.828{\left(1-2a/D\right)}^{2}+12.81{\left(1-2a/D\right)}^{3}-3.995{\left(1-2a/D\right)}^{4} \end{array}$$

where *D* is the diameter of the bar.

**Figure 6:**
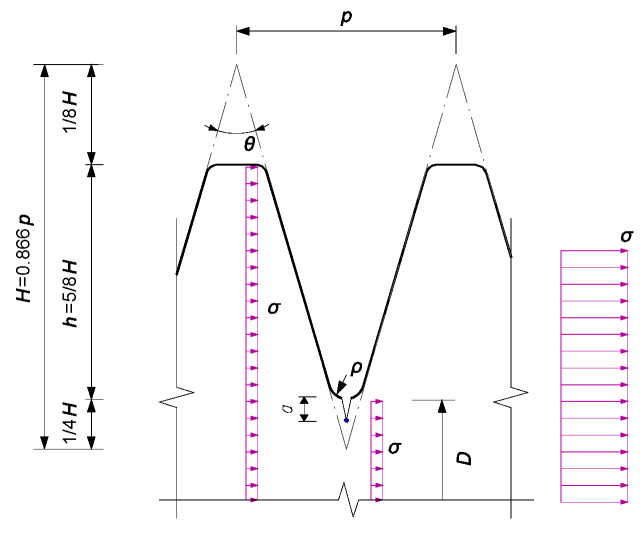
Schematic notation of an ISO metric screw thread with a root crack.

Afterwards, a two-dimensional loaded thread tooth-root model as shown in Figure 6 was suggested by Hasebe et al. [31] to represent a relationship between *K*_I_ and the thread depth or tooth height, *h*, and thread flank angle *θ*. SIF for an edge crack emanating from an angular corner in a semi-finite strip, *K*^θ^_I,SF_, is given by:

(12) $$K_{I,SF}^{\theta }=\sigma \sqrt{\pi a}\cdot 1.325{\left(a/h\right)}^{\left[-0.1867+0.0223\ln{\left(a/h\right)}^{2}\right]}e^{\left[H_{0}+H_{1}\ln(a/h)+H_{2}\ln{\left(a/h\right)}^{2}\right]}$$

where the constants are referred to based on curve fitting as *H*_0_ = (−0.138*θ*^3^ + 0.0064*θ*^4^ − 0.0001*θ*^5^)10^−6^, *H*_1_ = (0.3603*θ*^2^ − 0.0275*θ*^3^ + 0.0005*θ*^4^)10^−5^ and *H*_0_ = (0.1735*θ*^2^ − 0.0056*θ*^3^)10^−5^. Specifically, the SIF for a semi-finite strip with *θ* = 0 can be written as follows:

(13) $$K_{I,SF}^{0}=\sigma \sqrt{\pi a}\cdot 1.325{\left(a/h\right)}^{\left[-0.1867+0.0223\ln{\left(a/h\right)}^{2}\right]}$$

Alternatively, the SIF for a finite-thickness strip with *θ* = 0 can be taken as:

(14) $$K_{I,FS}^{0}=\sigma \sqrt{\pi a}\cdot \left[\begin{array}{l} 1.4-2.4\left(a/D\right)+10.1{\left(a/D\right)}^{2}+37.6{\left(a/D\right)}^{3}-196.3{\left(a/D\right)}^{4}+280.3{\left(a/D\right)}^{5}\\ -301.3{\left(a/D\right)}^{8}+981.7{\left(a/D\right)}^{13}-1545.7{\left(a/D\right)}^{17}+1140.5{\left(a/D\right)}^{23} \end{array}\right]$$

Therefore, the SIF for a finite thickness strip with *θ* can be obtained by a convention of *K*^θ^_I,SF_, *K*^0^_I,SF_, and *K*^0^_I,FS_ as:

(15) $$K_{I,FS}^{\theta }=K_{I,SF}^{\theta }K_{I,FS}^{0}/K_{I,SF}^{0}$$

The fracture mode of the test bolted spherical joint is characterised by the screw thread cracking of the bolt shank close to the contact surface between the bolted sphere and the sleeve. Regarding this, the stress concentration and the crack propagation at the roots of the screw threads are the further focus of the modelling. The profile of ISO metric screw thread (also known as standard thread) recommended by ISO 68-1 [32] is referred to and plotted in Figure 6. Each screw consists of a symmetric V-shaped thread with symmetrical neighbouring flanks, which is characterised by its height of the fundamental thread triangle, *H*, and its pitch, *p*. In the plane of the thread axis, the flanks of the V have an angle, *θ*, to each other, and the thread depth or tooth height *h* is equal to 5/8*H,* while the outermost 1/8 and the innermost 1/4 of the height of the V-shape are cut off from the profile of the fundamental thread triangle. Following the conversion of the trigonometric function, the relationship between the height of the fundamental thread triangle and the pitch can be found using *H* = 0.5*p*/tan(0.5*θ*). This can be further approximated as *H* = 0.866*p* when the thread flank angle, *θ*, is equal to 60° for ISO metric screw thread. However, it is worthy of note that the larger thread flank angle, i.e., *θ* = 80°, employed in steel conduit thread (Pg) metric threads as suggested by DIN EN 50,262 [33] is also taken into account for the analysis. On the other hand, the loading on the thread or tooth is different from many other analytical cases dealing with notch-type geometry, as documented in the literature [15], since the stress distribution area for the section of internal thread is reduced as compared to that of the external thread. As such, the internal screw thread root is characterised by the root radius, *ρ*, which can be adopted to further determine the notch sensitivity using *q* = 1/(1 + *ζ*/√*ρ*), and the Neuber’s constant for 10.9 metric grade can be obtained as *ζ* = 0.19√ mm [34].

## 4. Finite Element Modelling

**Figure 7:**
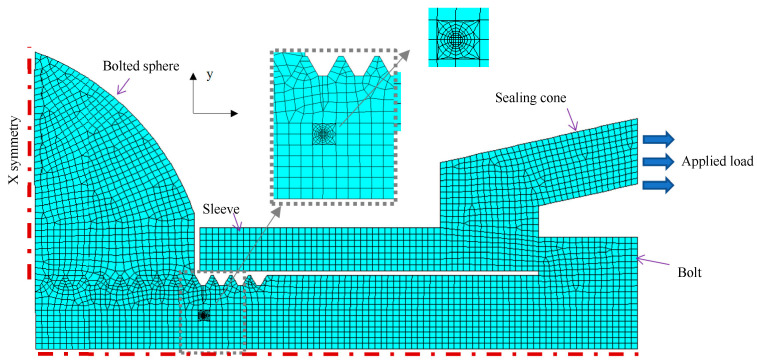
Illustration of the finite element model.

The finite element modelling for the prediction of SIF for the cracked threads of bolted spherical joints was performed using ANSYS software. A contrast is made between the results calculated from the above-mentioned theoretical calculation and modelling when bolted spherical joints with and without cracks at the screw thread root are concerned. The benchmark model was considered for an ISO metric screw-threaded bolt to a bolted sphere, in which the dimensions of the bolt are taken as: *D* = 24 mm, *H* = 2.598 mm, and *ρ* = 0.125 mm. The coarse pitch, i.e., *p* = 3 mm, is chosen as the commonly used default pitch for a given diameter. Due to symmetry, only a quarter of the bolted sphere and the high-strength bolt while a half of the sleeve and the sealing cone were created in a planar structure model, allowing for plane stress and strain states with symmetric restraints. For the fracture mechanics analysis, a surface crack was introduced at the root of the screw thread as corresponding to the profile plotted in Figure 6, and the region around the crack front was partitioned from the geometry of the thread to apply a very fine mesh in 2D analytical model analysis as shown in Figure 7. The crack body contains the singularity in front of the crack tip and an isoperimetric crack tip element so that all related stress concentrations can be properly simulated using an initial input file-aided processor. An 8-node plain stress element was used to mesh the crack region, and the rest of the model was meshed using bilinear four-sided meshing generated by the ANSYS pre-processor. The mesh size was refined near the crack region and the root of the screw thread region for accurate results, while relatively dense mesh was applied at the bolted sphere, the sleeve, and the sealing cone where a lower stress state was anticipated. For the sake of obtaining the optimum mesh size, a convergence study was performed, and the optimum number of elements around the region was noted to be nearly 200. The numerical SIFs were obtained using the displacement extrapolation method during post-processing, with the local crack tip coordinate initially established along the X axis while the Y axis is perpendicular to the crack face. The displacement components, *u* and ν, along the local Cartesian coordinate in crack faces are then defined as:

(16) $$\left\{\begin{array}{l} u\\ v \end{array}\right\}=\frac{1}{4G}{\left(\frac{r}{2\pi }\right)}^{1/2}\left\{\begin{array}{l} K_{I}\left[(2\chi -1)\cos\left(0.5\theta \right)-\cos\left(1.5\theta \right)\right]-K_{\mathrm{II}}\left[(2\chi -1)\sin\left(0.5\theta \right)+\sin\left(1.5\theta \right)\right]\\ K_{I}\left[(2\chi -1)\sin\left(0.5\theta \right)-\sin\left(1.5\theta \right)\right]-K_{\mathrm{II}}\left[(2\chi +3)\cos\left(0.5\theta \right)+\cos\left(1.5\theta \right)\right] \end{array}\right\}$$

where the constant *χ* = (3 − *υ*)/(1 + *υ*) with Poisson’s ratio, *υ* = 0.3 for steel material. The defined SIFs of the crack tip under a plane stress condition are then determined by inputting the KCALC command.

As shown in Figure 7, a quarter of the bolted sphere was symmetrically restrained in the *x* direction, while a half of the high-strength bolt was symmetrically restrained in the *x* direction as an application of the symmetry condition. Since only the presence of dry friction is accounted for at the junction between the high-strength bolt and the screw thread of the bolted sphere, the related Coulomb friction coefficient was defined as 0.1, while two contact conditions are considered: (1) between the threads of the bolted sphere and those of the high-strength bolt; and (2) between the inner face of the bolt head and its joined face of the sealing cone. The nominal tensile stress of *σ* = 100 MPa was applied at the end of the sealing cone to simulate the bending-induced tension load while it was transferred from its joined tubular member.

## 5. Results and Discussion

### 5.1. Experimental Results

**Figure 8:**
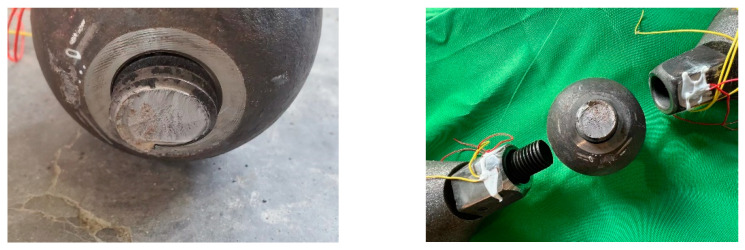
Fracture mode of test joint.

The typical fracture mode for the test specimens is the ultimate rupture of the high-strength bolt shank close to the contact surface between the bolted sphere and the sleeve, as shown in Figure 8. Visual inspection indicates that there is no apparent dispersion necking of the bolt shank and that the crack is propagating from the screw thread root to the bolt shank body. This can be anticipated since the screwing depth is sufficient to prevent premature pull-out and the tensile strength of the high-strength bolt is fulfilled, while the fracture of the bolted spherical joints is expected as a result of the stress concentration in bolt threads.

**Figure 9:**
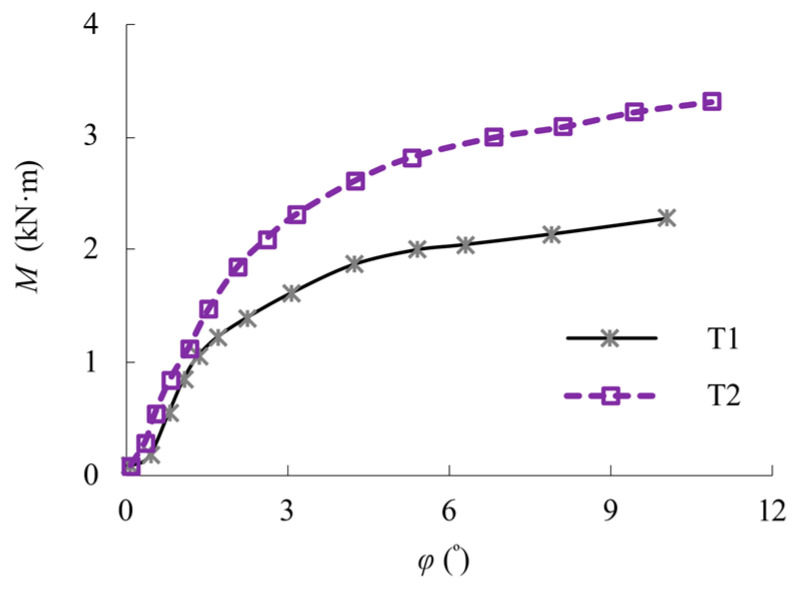
Comparison of *M*-*φ* curves.

All the experimental *M*-*φ* curves for the test bolted spherical joints are shown in Figure 9. The plots are arranged so that the results of *M* and *φ* are converted from these of the load applied and measured displacement, respectively, as mentioned in Section 3.1. Regrettably, the drops from the peak points of the test experimental *M*-*φ* curves were not caught due to some accidental data measurement at the final stage of loading. It can be seen that there are clear changes in the *M*-*φ* curves that deviate from proportionality to nonlinearity as an upper limit for the allowable stress that can be applied to the design flexural bending strengths of 1.2 kN·m and 1.75 kN·m for the test bolted spherical joints T1 and T2, respectively. Subsequent to this point, the test bolted spherical joints are shown to undergo a plastic deformation. Prior to the fracture, the test bolted spherical joints were demonstrated to have obvious flexural bending behaviour. The increase in bolt diameter has a notable influence on the increase in flexural bending capacity of the bolt-sphere joints, as the ultimate strength of the bolted spherical joints is increased by 46% when *d*_3_ is increased from 20 mm to 24 mm.

**Figure 10:**
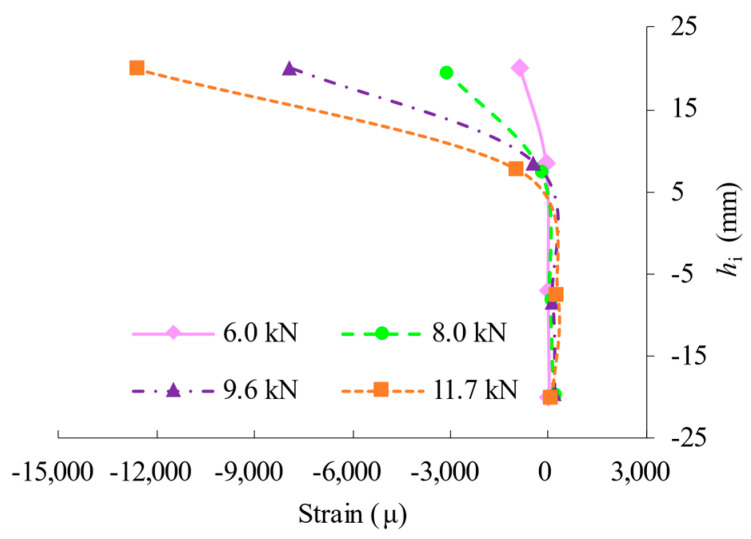
Comparison of load-strain curves of T2.

The load-strain curves of the sleeve of test specimen T2 are shown in Figure 10. There is no sleeve failure in the test scheme; however, the stress states of the sleeve are different above and below the axis passing through the centroid of the section of the tubular member. During the flexural bending, the upper parts of the sleeve suffer compression from the presence of a gap between the sleeve and the bolted sphere, which in turn produces a certain shift of the neutral axis of the section. Furthermore, the compressive strain of the ssleeve becomes prominent with the increase of the load to the maximum, i.e., the strain increasements amount to 59% and 302% when the applied load is amplified from 8 kN to 9.6 kN and 11.7 kN, respectively.

### 5.2. Numerical Verification

**Figure 11:**
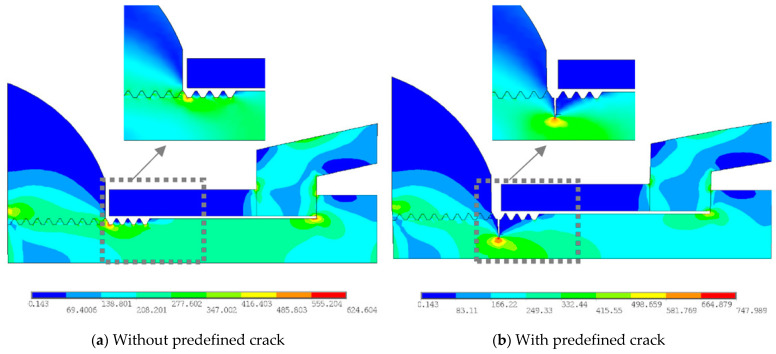
Typical stress distribution and fracture of bolted spherical joints (in MPa).

The Von Mises yield stress distributions for the bolted spherical joints without and with predefined cracks at the root of the screw thread are shown in Figure 11a and Figure 11b, respectively. In both cases, the sleeve without contact with the bolted sphere and the screw threads have the lowest stress distribution. The high-strength bolt resists the tensile load transferred from the sealing cone, so a relatively higher stress can be noted locally at the corner of the bolt head in connection with the bolt shank. Meanwhile, the bolted sphere is moderately stressed, with a maximum magnitude of 562 MPa, particularly in connection with the end screw threads. In contrast, the highest stresses, i.e., 624 MPa, can be found at the root of the first internal thread of the high-strength bolt outside the junction with the screw thread of the bolted sphere in the former case. This is in good agreement with an experimental observation of the fracture section of the bolt shank close to the contact surface between the bolted sphere and the sleeve, as shown in Figure 8.

For the latter case, the crack tip is greatly stressed to the maximum value of 747 MPa as the crack is propagated from the root of the first internal thread of the high-strength bolt outside the junction with the screw thread of the bolted sphere. In contrast, the aforementioned bolt head corner and the bolted sphere are much less stressed, with maximum magnitudes of 523 MPa and 499 MPa, respectively. This can be expected since the amplification of local stress at the root of the cracked first screw thread of the high-strength bolt occurs when the internal thread of the high-strength bolt and the external thread of the bolted sphere are mates and join together.

**Figure 12:**
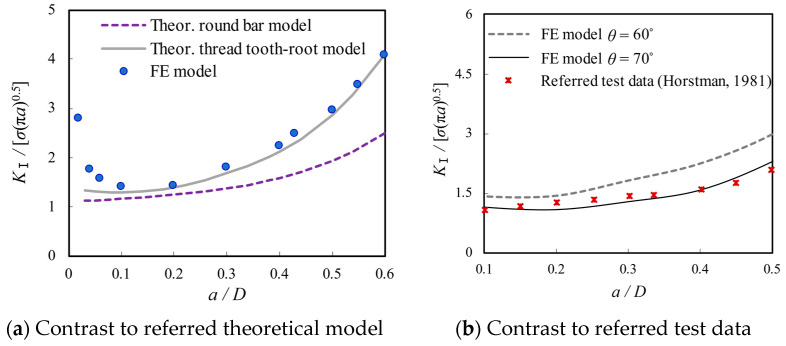
Illustration of verified results of the FE model with respect to *a*/*D* [35].

For the sake of further validating the developed finite element model, a parametric design language in ANSYS has been adopted to obtain the SIF results of the bolted spherical joints as the crack length is increased to instantaneous fracture of the bolt, i.e., *a* = 0.6*D*. Given that the analysis is regarded as a linear elastic problem with a homogeneous, isotropic material near the crack region, the SIFs for the crack tip in the plane stress state are calculated following the aforementioned KCALC command. The resultant numerical results of SIFs and the thread tooth-root model-based theoretical results of *K*^θ^_I,FS_ in Equation (15) are normalized with respect to *σ*(π*a*)^0.5^, which can be comparable to the round bar model-based theoretical results of *Y*(a) as dimensionless number geometric correction factor calculated by Equation (11). From a contrast of the theoretical and FE results of the dimensionless *K*_Ⅰ_ as shown in Figure 12a, it can be observed that the dimensionless *K*_Ⅰ_ from finite element modelling becomes much higher as *a*/*D* is reduced from 0.1 to 0, which is exactly opposite to the trend predicted by the thread tooth-root model-based theoretical results. This can be attributed to the local stress concentration effect induced by the profile of a screw with a V-shaped thread, which is underestimated by the theoretical expression. In other words, the dimensionless *K*_Ⅰ_ is supposed to be high, especially for very shallow cracks, and then decline to a minimum when *a*/*D* is increased to 0.1; subsequently, it is enlarged gradually with the further increase of *a*/*D*. Furthermore, the numerical results of the dimensionless *K*_Ⅰ_ are seen to be in good agreement with the thread tooth-root model-based theoretical results when the SIFs are gradually enlarged with *a*/*D* increasing from 0.1 to 0.6. The numerical results of the dimensionless *K*_Ⅰ_ are apparently underestimated by the round bar model-based theoretical prediction due to the neglect of the influence of screw thread details on SIFs. Similarly, it is indicated from a comparison in Figure 12b that the dimensionless *K*_Ⅰ_ from the referred test data in Ref. [35] related to single edge crack solid and hollow round bars loaded in tension is close to the FE model with *θ* = 70° but slightly lower than that with *θ* = 60° when *a*/*D* is approaching 0.5. Therefore, both the concentrated stress analysis without crack propagation and the SIF analysis with crack propagation are considered for the subsequent sensitivity parameter analysis.

### 5.3. Stress Amplification Influenced by Screw Thread Dimension

It is deemed necessary to first understand the local stress amplification of the root of the screw thread for the bolted spherical joints without predefined cracks or crack initialization influenced by the profile of the screw thread. According to literature [34], *ρ* and *θ* are the main factors influencing the SIFs of screw threads under a tensile stress field. Regarding this, the parameters are varied as 0.1, 0. 25, 0.5, 0.75, and 1 for *ρ* and 60°, 70°, and 80° for *θ,* representing the smoothness of the root and the steepness of the V-shape, which might further influence the local stress concentration. Meanwhile, all the other parameters are kept unchanged when the contrast is made. Von Mises stress, σ_Mises_, is used to characterise the numerical stress simulation of the bolted spherical joints.

**Figure 13:**
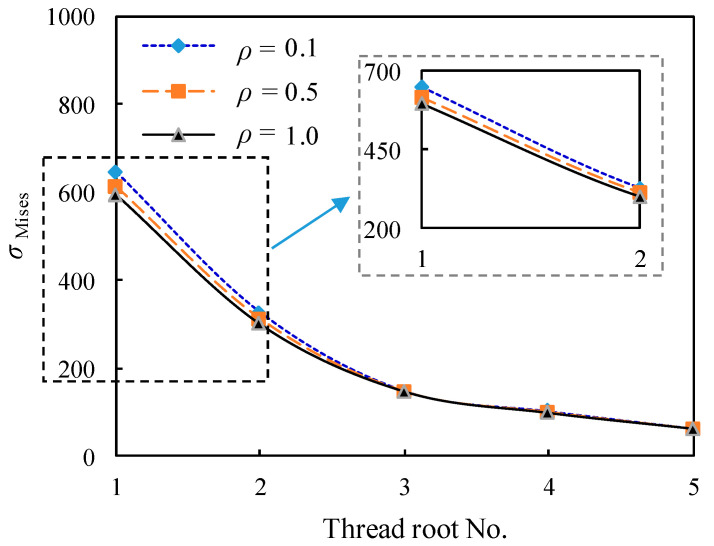
Comparison of the stress distribution at the numbered screw threat root.

Firstly, only the screw thread root radius *ρ* is varied in each simulation scheme, and all bolted spherical joints are assumed to have the internal thread of the high-strength bolt and the external thread of the bolted sphere positively locked and sealed threaded assemblies, preventing axial movement. Considering the profile of an ISO metric screw thread (*p* = 3 mm, *θ* = 60°) with an M24 bolted spherical joint as an example to describe the modelling results. The comparison of the stress distribution from the root of the first internal thread of the high-strength bolt outside the junction with the screw thread of the bolted sphere to those related to consecutive four threads inside the bolted sphere under tensile traction is shown in Figure 13. It can be generally observed that the stress at the root of the internal thread of the high-strength bolt is reduced gradually from the first screw thread root (No. 1) to the innermost bolted sphere in sequence from No. 2 to No. 5, corresponding to nearly 49%, 77%, 84%, and 90% reduction of *σ*_Mises_, respectively. Moreover, the stress concentration of the bolted spherical joint influenced by the screw thread root radius is discernible since *σ*_Mises_ is decreased by 5% and 8% when *ρ* is increased from 0.1 to 0.5 and 1.0, respectively, for the first screw thread root. However, such a decrease becomes less obvious with the increase of the individual thread root number, i.e., the deeper screw thread root inside the bolted sphere joining the internal thread of the high-strength bolt.

**Figure 14:**
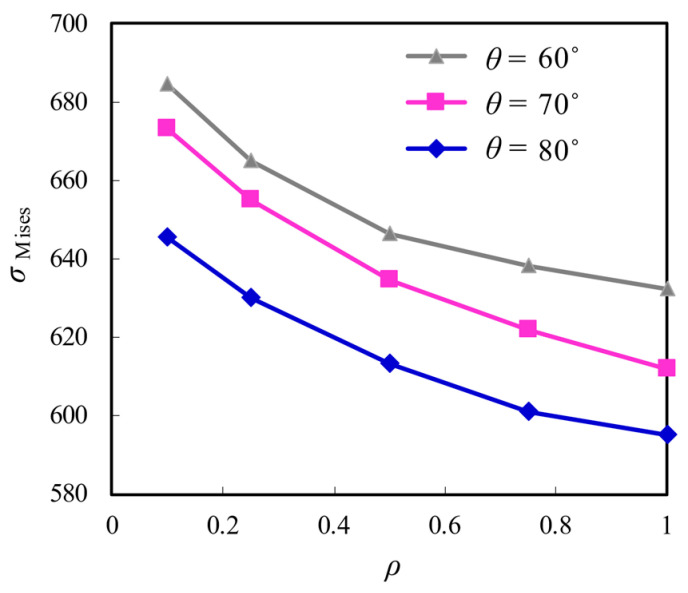
Comparison of the stress distribution influenced by ρ and θ.

Based on the above comparison, only the Von Mises stress at the root of the first internal thread of the high-strength bolt outside the junction with the screw thread of the bolted sphere as a maximum is considered for the following analysis. The comparison of *σ*_Mises_ influenced by the screw thread root radius and the thread flank angle is displayed in Figure 14. It can be seen that *σ*_Mises_ for the larger *θ* are considerably low. For example, *σ*_Mises_ for *θ* = 70° and *θ* = 80° are reduced by 3% and 6%, respectively, when compared to *σ*_Mises_ for *θ* = 60°. Although the decrease of *σ*_Mises_ with the increase of *ρ* from 0.1 to 0.5 replicates a similar trend for all contrast cases, a slightly larger decrease of *σ*_Mises_ for the bolted spherical joints with lager *θ* as *ρ* is further 0.1 to 0.5. For example, the reduction of *σ*_Mises_ is nearly 8% and 10% for the joints with *θ* = 70° and 7% and 11% for the joints with *θ* = 80° when *ρ* is correspondingly increased to 0.8 and 1.0. Additionally, it is noted that *σ*_Mises_ and its resultant stress concentration can be greatly reduced with combined amplification of *ρ* and *θ*, e.g., *σ*_Mises_ for *ρ* = 1 and *θ* = 80° is only 85% that for *ρ* = 0.1 and *θ* = 60°.

### 5.4. Stress Amplification Influenced by Screw Thread Dimension

The work in the previous section indicates that *ρ* and *θ* have certain influences on the local stress amplification of the bolted spherical joints without crack initiation. However, it seems that both factors are not properly accounted for in the referred theoretical models for the calculation of SIFs. Hence, it is worth exploring the SIFs of cracked bolted spherical joints through parametric sensitivity analysis with several factors considered comprehensively, including the variety of *ρ* (0.1, 0.25, 0.5, 0.75, and 1), *θ* (60°, 70°, and 80°), and *p* (2, 2.5, and 3). Moreover, the corresponding design parameters for the crew thread can be converted for *H* (1.19, 1.35, 1.43, 1.49, 1.79, 2.14, 2.17, and 2.6) and *h* (0.75, 0.89, 0.93, 1.08, 1.12, 1.34, 1.35, and 1.62), as listed in Table 3. In this study, the benchmark model was considered as a basic example for a contrast, and then a parametric study including design variables in the SIF analytical model was performed to explore design alternatives influencing the mentioned bolted spherical joints with crack propagation at the root of the screw thread. It was also done by adjusting a fixed variable listed below when all other variables remain unchanged, and then the changes in such a fixed variable can be quantified properly.

#### 5.4.1. Effect of *ρ* on SIFs

**Figure 15:**
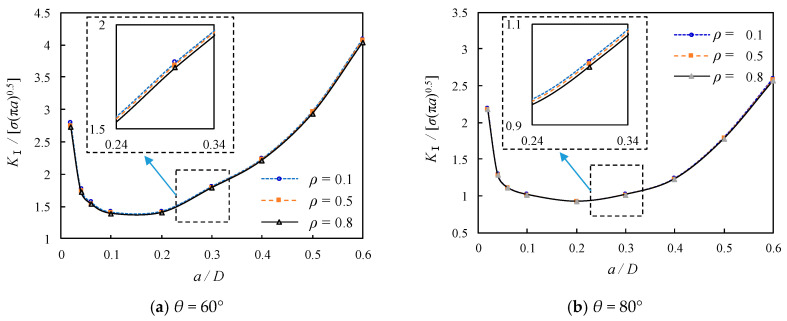
Relation between dimensionless SIFs and *a*/*D* for joints with varying *ρ*.

The profile of each screw is featured by a symmetric V-shaped thread connecting with each other with the root arc, which can also be regarded as a notch and defined by the root radius. According to the aforementioned reference to the notch sensitivity, *q* is obviously changed with different *ρ*, as listed in Table 3. However, its certainty for the evaluation of the local stress concentration was not confirmed in Ref. [34], and therefore the suitability in the appraisal of SIFs of bolted spherical joints with predefined cracks is also unknown. Figure 15 shows the comparison of the effect of *ρ* on the dimensionless SIFs for the cracked threads of bolted spherical joints with *θ* = 60° and 80°. A general trend can be seen that the dimensionless *K*_Ⅰ_ decreased first as *a*/*D* was between 0.1 and 0.2 and then increased afterwards for all cracked bolted spherical joints, irrespective of different *ρ* and *θ*. Moreover, the root radius is observed to have very little effect on the dimensionless *K*_Ⅰ_ of the cracked threads of bolted spherical joints since the curves for different *ρ* are almost coincident for each comparison group. On an expanded scale with regions associated with the range in the vicinity around *a*/*D* = 0.3, it can be identified that the increase of *ρ* resulted in a reduced dimensionless *K*_Ⅰ_. For example, the dimensionless *K*_Ⅰ_ for the cracked threads of bolted spherical joints with *θ* = 60° and 80° is decreased by 2.3% and 2.9%, respectively, when *ρ* is increased from 0.1 to 1.0. These listed decreases can be neglected not only when compared with the amplification of stress concentration as indexed by the aforementioned notch sensitivity, i.e., *q* is increased by 34.5% when *ρ* is increased from 0.1 to 1.0, but also when compared with the decreased *σ*_Mises_ results as discussed in the foregoing section. Alternatively, the observation of the finite element modelling in Figure 15 seems to correlate well with that reported in Ref. [36], in which root radius effects are nearly attenuated when *a*/*D* is greater than 0.015. Therefore, it can be concluded that the effect of *ρ* is negligible on the SIFs of the bolted spherical joints, with not very shallow crack propagation at the root of the first internal thread of the high-strength bolt outside the junction with the screw thread of the bolted sphere.

#### 5.4.2. Effect of *p* on SIFs

**Figure 16:**
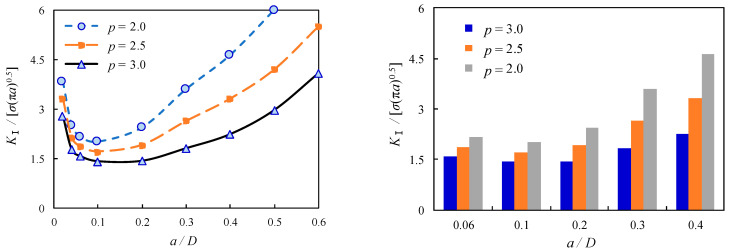
Relation between dimensionless SIFs and *a*/*D* for joints with varying *p*.

The dimension of the pitch, *p*, usually varies with the thread depth or tooth height, (*h*). Compared to the coarse pitch, which is the commonly used default pitch in crew threads, fine crew threads also have a greater minor diameter than coarse crew threads, which means the bolt has a greater cross-sectional area for the same nominal diameter and therefore a greater load-carrying capacity. Most reported applications of the analytical equation of Equation (14) are unable to find appropriate SIF solutions allowing for *p* as a variable. Hence, the range of *p* varied as 2.0, 2.5, and 3.0, representing the variation from fine screw threads to coarse screw threads, is considered in the finite element modelling. Figure 16 shows the curve diagram and bar chart representing the general trend and numeric dimensionless *K*_Ⅰ_ values for levels of categorical crack propagation for the effect of *p* on the dimensionless *K*_Ⅰ_ of the cracked threads of bolted spherical joints. It can be seen that the dimensionless *K*_Ⅰ_ is gradually decreasing with the increase of *p* as the crack propagates from the root of the first internal thread of the high-strength bolt outside the junction with the screw thread of the bolted sphere. Such a trend becomes rather pronounced when the dimensionless *K*_Ⅰ_ steps into consistent elevation from the minimum and *a*/*D* is larger than 0.1, which can be attributed to greater stress concentration induced with greater crack propagation and even fracture appearance, as shown on the left side of Figure 16. To further quantify such an elevation, it can be seen from the right side of Figure 16 that when *p* is decreased from 3.0 to 2.0, the dimensionless *K*_Ⅰ_ are increased by 36.7% and 41.5% for *a*/*D* = 0.06 and *a*/*D* = 0.1, respectively, while the dimensionless *K*_Ⅰ_ are further increased by 70.6%, 98.9%, and 106.7% for *a*/*D* = 0.2, *a*/*D* = 0.3, and *a*/*D* = 0.4, respectively. It can be concluded that *p* has a notable effect on the intensity of the stress field and the stresses in the crack tip region of the bolted spherical joints, especially for the extended crack propagation beyond *a*/*D* = 0.1. Moreover, bolted spherical joints containing fine screw threads are likely to achieve better static load-carrying capacity but attenuate fracture resistance when compared to those containing coarse screw threads.

#### 5.4.3. Effect of *θ* on SIFs

**Figure 17:**
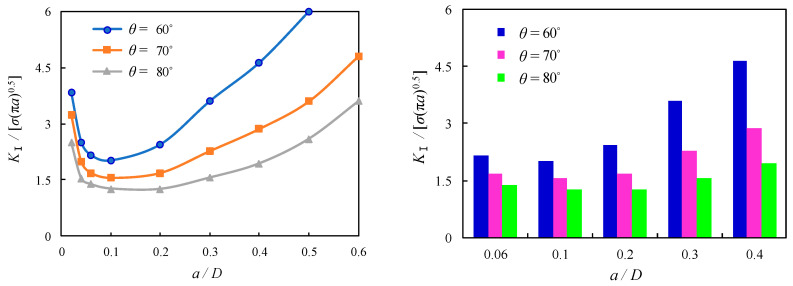
Relation between dimensionless SIFs and *a*/*D* for joints with *p* = 2.

**Figure 18:**
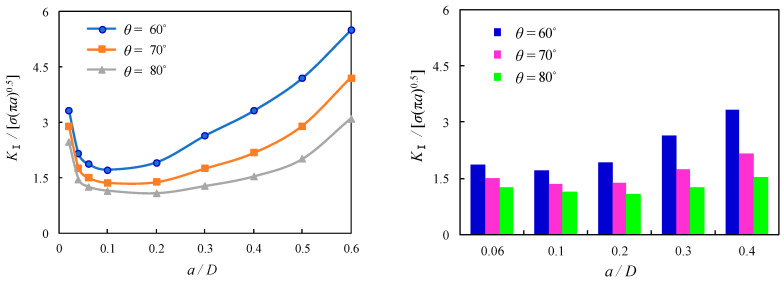
Relation between dimensionless SIFs and *a*/*D* for joints with *p* = 2.5.

**Figure 19:**
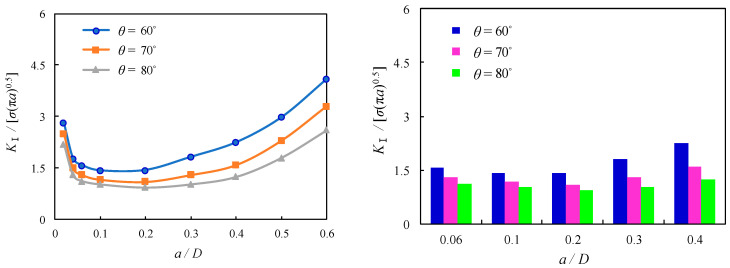
Relation between dimensionless SIFs and *a*/*D* for joints with *p* = 3.

The thread flank angle is the angle between neighbouring flanks having identical angles for ISO metric screw threads. The influence of thread flank angle on the local stress magnitude was found for bolted spherical joints without predefined cracks, as mentioned in the foregoing section, while its influence on SIFs was also found for bolted spherical joints with shallow cracks, as reported by Hasebe [31]. However, the thread flank angle is not an independent variable since the height of the fundamental thread triangle and its converted thread depth, or tooth height, is also subject to change due to their variation. For the sake of a further comparison of such an effect on the dimensionless *K*_Ⅰ_ of the cracked threads of bolted spherical joints, *θ* is varied as 60°, 70°, and 80° in the FE solutions. The dimensionless *K*_Ⅰ_ is shown to be generally reduced with the increase of *θ* as a general variation trend with the variation of related parameters. As compared among Figure 17, Figure 18 and Figure 19, the reduction of dimensionless *K*_Ⅰ_ is similar and approximated as 18% and 29% for the increase of *θ* from 60° to 70° and 80°, respectively, when *a*/*D* is varied between 0.06 and 0.1. In contrast, more discernible variation can be seen for the effect of *θ* on the dimensionless *K*_Ⅰ_ with the superimposed variable *p,* which is also expected to be a relevant parameter for the profile of the screw with crack propagation greater than *a*/*D* = 0.1. As an example, for the joints with *p* = 2, when *θ* is increased from 60° to 70° and 80°, the corresponding reductions of dimensionless *K*_Ⅰ_ are nearly 44% and 93% for *a*/*D* = 0.2, respectively, and nearly 61% and 138% for *a*/*D* = 0.4, respectively, as shown in Figure 17. In contrast, for the joints with *p* = 2.5, the corresponding reductions of dimensionless *K*_Ⅰ_ are nearly 38% and 78% for *a*/*D* = 0.2, respectively, while nearly 52% and 116% for *a*/*D* = 0.4, respectively, as shown in Figure 18. For the joints with *p* = 3, the corresponding reductions of dimensionless *K*_Ⅰ_ are nearly 30% and 53% for *a*/*D* = 0.2, respectively, while nearly 40% and 80% for *a*/*D* = 0.4, as shown in Figure 19. It can be concluded that the increase of *θ* has a notable effect on the decrease of SIF for the cracked threads of bolted spherical joints since the increased crack propagation can be moderately alleviated with decreased steepness of the flank thread.

## 6. Concluding Remarks

This paper presents experimental and FE modelling results of bolted spherical joints subjected to flexural bending-induced fracture. The aim of this research is to better understand the capacity related to the overall fracture section and the deterioration induced by the crack in screw threads. The experimental results are given in terms of the fracture mode, the moment versus rotation relation, and the load versus strain relation. The finite element modelling is presented in terms of the stress amplification and stress concentration influenced by screw thread dimensions. Based on the experimental and finite element modelling results, the following conclusion can be drawn.

The section close to the contact surface between the bolted sphere and the sleeve had a substantial impact on the fracture of the bolted spherical joints, even if the screwing depth is sufficient to prevent the bolt from pulling out. Prior to the facture, the bolted spherical joints are demonstrated to have obvious flexural bending behaviour whose strength is greatly influenced by the bolt diameter. The upper parts of the sleeve suffer from compression due to the presence of a gap between the sleeve and the bolted sphere, which in turn produces a certain shift of the neutral axis of the section. Accordingly, a theoretical expression allowing for a heightened neutral axis was proposed and shown to achieve good accuracy for predicting the flexural bending capacity of the fracture section. Based on the finite element modelling, the stress magnitude at the root of the screw thread can be greatly enhanced with a smaller thread root radius and flank angle. Moreover, the increase in thread root radius, in contrast, is not a critical parameter in the reduction of stress concentration factors for the bolted spherical joints. The general trend of the variation of stress intensity factors is not consistent or linear but decreases first as the crack propagates to 10%~20% of the bolt diameter and then increased afterwards until the fracture of the bolt screw thread. The resultant stress intensity factors based on validated finite element modelling are demonstrated to be underestimated by the referred round bar model in theory without allowance of screw thread details. Similar underestimation is also evident when the stress intensity factors of single-edge crack solid and hollow round bars loaded in tension are compared. Among other design parameters, the enlarged pitch thread is a dominant player in reducing stress intensity factors by 71~107% when the crack is propagated to 20~40% of the bolt diameter. In addition to the increased pitch, the stress intensity factors are reduced further 80~138% with the application of relief thread angle. Therefore, the moderate steepness of the flank thread is recommended for bolted spherical joints concerned by fracture resistance, even if the fine screw threads may be employed to achieve good static load-carrying capacity. The results and observations from this work, based on limited experiments and parametric studies, build a good reference for future development of design guidelines for bolted spherical joints.

## Figures and Tables

**Table 1:** Dimension and flexural moment of test specimens.

Test No.	Dimension (mm)								*M* (kN·m)		
	*d*_1_	*d*_2_	*d*_3_	*d*_4_	*d*_5_	*d*_6_	*d*_7_	*d*_8_	*M*_test_	*M*_pred_	*M*_test_/*M*_pred_
T1	50	36	20	78	18	45	77	400	1.20	1.14	1.05
T2	50	43	24	82	20	60	87	400	1.75	1.73	1.01

**Table 2:** Mechanical properties of connection materials.

Component	Elastic Modulus (GPa)	Yield Stress (MPa)	Ultimate Stress (MPa)	Elongation (%)
High-strength bolt	206	936	1040	9
bolted sphere, sleeve, end sealing cone	206	390	591	35

**Table 3:** Geometric range of screw threads for bolted spherical joints.

*a*/*D*	Basic Geometric Parameters			Design Parameters		
	*ρ* (mm)	*p* (mm)	*θ*	*H* (mm)	*h* (mm)	*q*
0.04~0.6	0.1, 0.25, 0.5, 0.75, 1	2, 2.5, 3	60°, 70°, 80°	1.19, 1.35, 1.43, 1.49, 1.79, 2.14, 2.17, 2.6	0.75, 0.89, 0.93, 1.08, 1.12, 1.34, 1.35, 1.62	0.62, 0.78, 0.84

## Data Availability

All data generated or used during the study are available from the corresponding authors upon request.
